# Draft Genome Sequence of a *Cardiobacterium hominis* Strain Isolated from Blood Cultures of a Patient with Infective Endocarditis

**DOI:** 10.1128/genomeA.00999-16

**Published:** 2016-09-22

**Authors:** Florian Tagini, Trestan Pillonel, Sandra Asner, Guy Prod’hom, Gilbert Greub

**Affiliations:** a Laboratory Department, Institute of Microbiology, University of Lausanne and University Hospital Center, Lausanne, Switzerland; b Pediatric Infectious Diseases and Vaccinology Unit, Department of Pediatrics, University Hospital Center, Lausanne, Switzerland; c Department of Internal Medicine, Service of Infectious Diseases, University Hospital Center, Lausanne, Switzerland

## Abstract

*Cardiobacterium hominis* is a well-known commensal bacterium of the oral cavity and an agent of infective endocarditis in humans. Here, we provide a draft genome sequence of a pathogenic strain isolated from blood cultures of a patient with infectious endocarditis.

## GENOME ANNOUNCEMENT

*Cardiobacterium hominis*, which is encountered in the oral and nasal floral (1), is also a member of the HACEK group (*Haemophilus* spp., *Aggregatibacter* spp., *Cardiobacterium hominis*, *Eikenella corrodens*, and *Kingella* spp.). HACEK group bacteria are fastidious Gram-negative causative agents of infective endocarditis.

The sequenced strain was isolated from blood cultures drawn from a 4-year-old child hospitalized at the University Hospital of Lausanne, Switzerland. The patient presented with fever, weakness, and a history of complex congenital cardiopathy, which required extensive cardiac surgery and the insertion of prosthetic material 3 years prior to the event. He was successfully treated with ceftriaxone for 6 weeks and gentamicin for 3 weeks, and the infected cardiac prosthetic material was removed.

Genomic DNA was extracted using the protocol for Gram-negative bacteria with the Wizard Genomic DNA purification kit (Promega). Sequencing was performed using the MiSeq desktop sequencer (Illumina) to produce 150-bp paired-end reads. Reads shorter than 150 bp were removed using Trimmomatic version 0.35 (2). *De novo* assembly was done using SPAdes genome assembler version 3.6.2 (3) with different *k*-mer values ranging from 43 to 127. Best assembly was chosen using Quast version 3.2 (4), based on the best *N*_50_ and *L*_50_ scores and lowest number of contigs larger than 1,000 bp. Annotation was performed using the RAST system version 2.0 (5) and browsed with SEED viewer (6). Finally, we searched for specific antibiotic resistance genes with ResFinder version 2.1 (7) and for prophage sequences with PHAST (8). Identity between translated coding sequences and corresponding proteins in the NCBI database was determined using CLUSTAL-W (9).

Sequencing produced a total of 2,456,795 150-bp paired-end reads that were assembled into 114 contigs larger than 300 bp. After removing contigs that were (i) smaller than 1,000 bp and (ii) could not map against the currently available genome on NCBI, the final assembly size is 2,646,022 bp with a G+C content of 59.01%. The RAST system predicted 2,489 CDSs. Among them, 110 are possibly involved in cell wall metabolism, 57 in virulence and defense, 205 in carbohydrates metabolism, 188 in protein metabolism, 89 in lipid metabolism, 120 in RNA metabolism, and 96 in DNA metabolism.

The annotation revealed nine putative CDSs for multidrug resistance efflux pumps possibly involved in various antibiotic resistances. However, Resfinder did not identify any other resistance genes, and we observed a high sensitivity to ceftriaxone and gentamicin by testing the antibiotic susceptibility. In addition to 19 pili-associated CDSs, we noticed a CDS sharing 59.18 to 88.58% identity with hypothetical proteins of other *Cardiobacterium* spp. and 45.89% identity with a component protein of the adhesin complex of *Eikenella corrodens*, another agent of infective endocarditis. Finally, we found one complete and two incomplete prophage sequences.

We hope that this draft genome sequence will be helpful for further genomic comparisons aiming to better understand *C. hominis* pathogenic features.

### Accession number(s).

The annotated genome sequence of *C. hominis* strain CHUV0807 has been deposited in the DDBJ/EMBL/GenBank database under the accession number FKLO01000000. 
